# Effects of Pharmacist Intervention on Community Control of Hypertension: A Randomized Controlled Trial in Zunyi, China

**DOI:** 10.9745/GHSP-D-20-00505

**Published:** 2021-12-31

**Authors:** Ying Li, Guoqin Liu, Chaojie Liu, Xianhong Wang, Yalin Chu, Xiaoqin Li, Wenhao Yang, Yewei Shen, Fang Wu, Wenzhi Zhang

**Affiliations:** a School of Management, Zunyi Medical University, Zunyi, People's Republic of China.; b The Second Affiliated Hospital of Zunyi Medical University, Zunyi, People's Republic of China.; c School of Psychology and Public Health, La Trobe University, Melbourne VIC 3086, Australia.; d Department of Pharmacy, Affiliated Hospital of Zunyi Medical University, Zunyi, People's Republic of China.

## Abstract

**Objective:**

We aimed to test the effects of pharmacist intervention on the community control of hypertension through a comparative randomized controlled trial.

**Methods:**

We recruited adult hypertensive patients with comorbidity or confusion with medication (n=636) from 2 community health centers in Zunyi, China. They were randomly and equally divided into 2 groups. Both groups received the usual care and participated in the community systematic management program of hypertension. Participants in the intervention group were given interventions from pharmacists, including a monthly review of medications, patient education, and medication adjustment advice to medical doctors over 6 months. Participants' blood pressure was assessed at baseline, 3 months, and 6 months. Participants' knowledge and medication adherence were measured using a questionnaire before and after the trial.

**Results:**

Compared to the control group (n=298), a significantly higher percentage of participants in the intervention group (n=290) had their blood pressure under control 3 months (46.9% vs. 38.3%, *P*=.034) and 6 months (60.7% vs. 40.9%, *P*<.001) after the interventions. Difference-in-differences analyses showed that the pharmacist intervention resulted in an increase in knowledge scores by 12.55 points (*P*<.001), a decrease in systolic blood pressure by 6.65 mmHg (*P*=.001), and a decrease in diastolic blood pressure by 7.26 mmHg (*P*<.001) compared to the controls after adjustment for variations in potential confounding factors. The odds of participants passing the hypertension knowledge tests in the intervention group was 4.45 times those in the control group (*P*<.001). Similarly, it was found that the intervention group had higher odds of not needing any medication adjustments (adjusted odds ratio [AOR]=2.75, *P*<.001) and having their blood pressure under control (AOR=2.18, *P*=.002) compared to the control group.

**Conclusion:**

It is evident that pharmacist intervention has significant short-term effects on improving the knowledge and medication adherence of hypertensive patients, as well as timely medication adjustments from medical doctors, resulting in lowered blood pressure and an increased control rate. Further studies should explore the long-term sustainability of the effects of community pharmacist intervention.

## Abstract (precis)

There has been growing interest in the role of pharmacists in managing chronic conditions. We tested the effects of a pharmacist intervention on community control of hypertension. Findings showed significant short-term improvement in patient knowledge, medication adherence, and lowered blood pressure.

## Teaser

**Key Finding:**

- The results from this randomized controlled trial of a pharmacist intervention, which included patient education and medication, are encouraging, showing significant short-term (at 3–6 months) improvement in blood pressure control, patient knowledge on hypertension, and medication adherence.

**Key Implications:**

- Community pharmacists can contribute to better management of chronic conditions, such as hypertension, as part of an interdisciplinary team in an integrated care network to improve the continuity and coordination of patient care.
- The findings shed some new insight into the unique role that pharmacists can play by working in partnership with the health care providers, patients, and other community health workers.

## INTRODUCTION

Hypertension is the most common chronic disease and a primary risk factor for cardiovascular and cerebrovascular diseases. Although effective treatment regimens exist for hypertension that can significantly reduce mortality and prevent the development of complications,^1^ the control of blood pressure has remained a great challenge. The World Health Organization (WHO) estimated that 1.13 billion people in the world have hypertension.^2^ But more than four-fifths have failed to bring their blood pressure under control, resulting in 10 million deaths every year.^3^ Hypertension also brings substantial financial burdens to the world, which affect low- and middle-income countries (LMICs) disproportionately. About two-thirds of hypertensive patients in the world live in China.^2^ A study found that hypertension treatments account for 6.61% of the total health expenditure in China.^4^

There are many underlying reasons for the poor control of blood pressure, of which poor knowledge of patients and nonadherence to prescribed therapy are deemed critical contributing factors.^5^^–^^7^ It is widely believed that optimizing drug treatment is the key to achieving the successful control of blood pressure.^5^^,^^8^^,^^9^ A systematic review and meta-analysis showed that 83.7% of cases of uncontrolled hypertension are a result of nonadherence to drug therapy.^7^ But unfortunately, there is no simple solution. Many efforts have been made to address this issue, ranging from patient education and guidelines on professional practice to technological innovations.^10^ Empirical evidence has highlighted the importance of cross-disciplinary collaboration and partnerships between health care providers and patients.^11^

In recent years, interest has grown in enhancing pharmacist services in primary care for improved management of hypertension. Pharmacist services have shifted focus from ensuring drug supply to providing patient-centered care. Studies conducted in high-income countries, such as the United States and Canada, have demonstrated that pharmacist interventions on hypertension can help achieve remarkable results,^12^^–^^14^ including improved adherence to drug therapy, lowered blood pressure, better health outcomes, and savings in relation to medical expenditure.^15^^–^^20^ Consequently, the Canadian^8^^,^^21^ government has issued guidelines and standards for community pharmacist care for hypertensive patients. As a highly skilled profession, community pharmacists have performed well in monitoring chronic conditions, educating patients, and providing medication consultations to medical doctors and patients.^11^^,^^15^^,^^22^

The focus of pharmacist services has shifted from ensuring drug supply to providing patient-centered care.

However, there is a dearth of evidence on the effectiveness of community pharmacist interventions on hypertension in LMICs. In a systematic review on the impact of interventions by community pharmacists on the control of hypertension, Cheema et al.^23^ identified only 5 of 16 randomized controlled trials conducted in LMICs. There is a consensus that community-oriented primary care is the most appropriate setting for managing hypertension, which requires continuous and coordinated care.^24^ In high-income countries, pharmacists are usually more easily accessible in the community than medical doctors, giving community pharmacists an advantage in managing hypertension. But this is not necessarily the case in LMICs where a shortage of properly trained pharmacists is common.^25^

This study fills the gap in the literature by testing the impact of pharmacist interventions on hypertension in primary care facilities in China. Since 2009, the Chinese government has increased its investment in primary care dramatically. A total of 9,352 (in 2018) community health centers were developed over a short period, covering the entire population.^26^ In line with the international evidence, community health care services have been shown to serve as a major force in managing chronic conditions. However, workforce development in community health care services has been focused on general practice and community nursing. Little attention, if any, has been paid to the development of community pharmacists although all community health care services dispense medications. The outcome of the primary care reform in China has attracted serious debate.^27^ It appears that the management programs for chronic diseases have been far from successful. In 2018, only 30% of hypertensive patients received antihypertension treatments,^28^ well below the level (40%–80%) of high-income countries such as Canada, Australia, the United Kingdom, the United States, and Germany.^29^ The community management of chronic diseases is delivered in China free of charge by a general practice team as part of the essential public health service package.^30^^,^^31^ High workloads of the general practice team and a lack of attention to medication adherence have often been blamed for the unsatisfactory outcome.^4^^,^^32^^,^^33^ Indeed, community health care services have been struggling to attract medical doctors. In China, about 1.53 million medical doctors (2.5 per 1,000 population) work in primary care, accounting for 39% of the medical workforce.^34^ Adding to the complexity is the low qualifications of primary care medical workers (31% do not have a medical degree),^35^ the high prevalence of irrational prescriptions,^28^ and the self-medication of consumers.^36^ As a result, there have been increasing calls recently from both researchers and policy makers to explore the role of community pharmacists.

## MATERIALS AND METHODS

### Study Setting

A comparative randomized controlled trial was conducted in 2 urban community health centers in the Zunyi municipality of Guizhou province in China. The trial was registered with the Chinese Clinical Trials Registry (registration number: ChiCTR1900028368).

Zunyi has a population of more than 6 million. In 2018, it had an average gross domestic product (GDP) of US$5,656 per capita,^37^ about half of the national average in mainland China.^38^ According to the World Bank, Zunyi is ranked at the lower end of middle-income economies.^39^

Community health services were developed in China as a hub for the delivery of primary health care services, covering essential medical services, disease prevention and control, care for vulnerable populations, rehabilitation, family planning, health education, and health promotion. Each community health center was designated to cover a residential community ranging in number from 20,000 to 60,000 people. Zunyi has 19 urban community health centers.^37^ In 2018, a total of 822,600 patients sought medical attention from these health centers.^40^

Two medium-sized urban community health centers were selected from the Huichuan district, the economic, political, and cultural center of Zunyi. Huichuan was ranked 48 among the 219 economic and technological development zones in China.^41^ A total of 6,500 hypertensive patients registered with the 2 centers over the past 9 years.

### Study Participants

Eligible participants of this study were those aged 18 years or older who had a confirmed diagnosis of hypertension. They had enrolled in the community systematic management program for hypertension and received relevant services. The participants also had to meet at least 1 of the following criteria: (1) taking antihypertensive medications; (2) having coexisting chronic conditions such as diabetes; (3) reporting confusion with their own medication regimen; or (4) missing medicines frequently. Pregnant and lactating women and patients with recorded cognitive impairments were excluded from this study.

**FIGURE 1:**
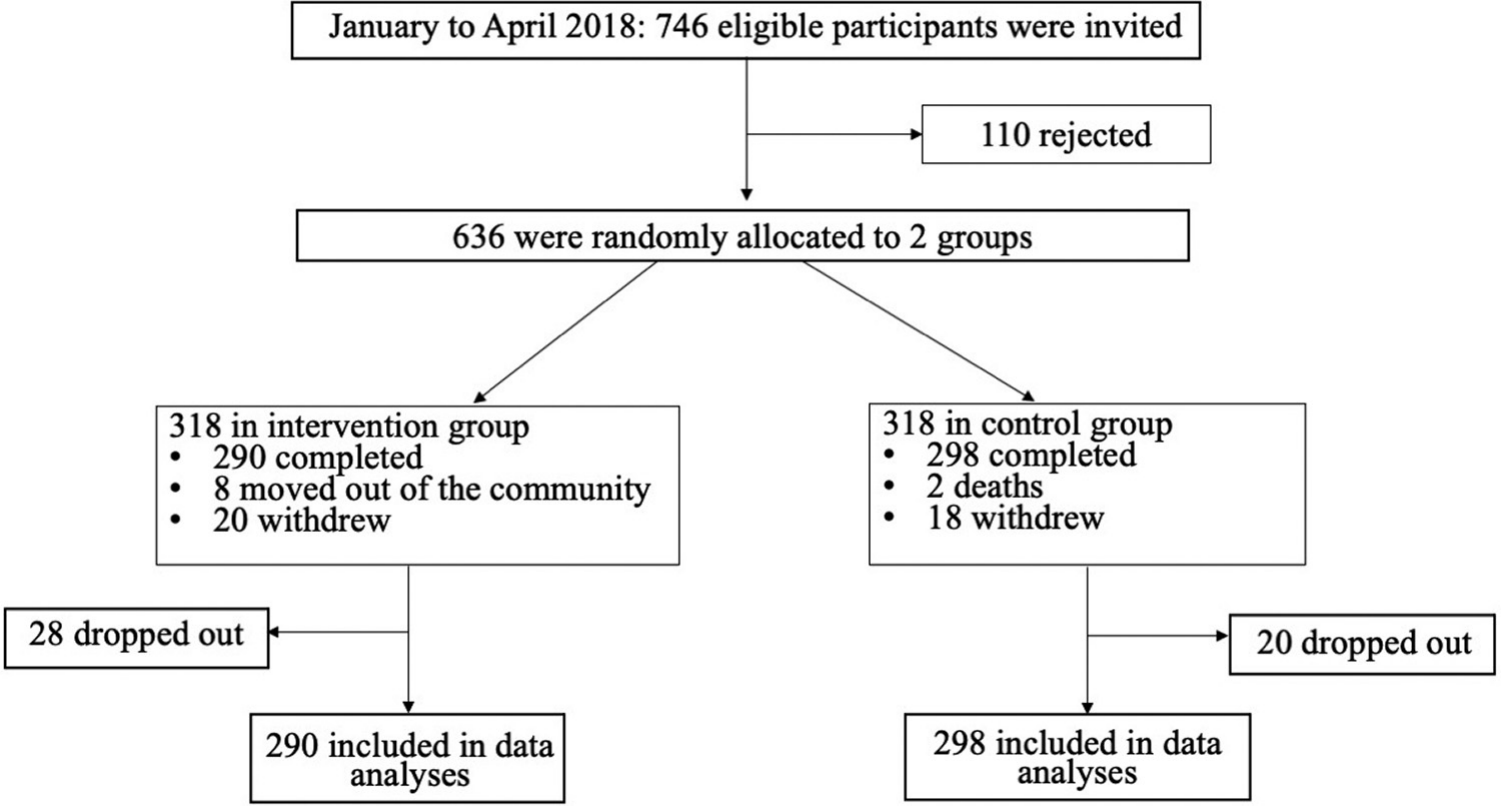
Flow Chart of Participant Recruitment in a Randomized Controlled Trial on Effect of Pharmacist Interventions on Management of Hypertension, China

Eligible patients who sought medical attention for hypertension from the 2 community health centers from January to April 2018 were invited by their doctors to participate in this study. In total, 746 were invited and 110 rejected. This resulted in a sample size of 636 participants, who were randomly allocated into the control and intervention groups equally. Eventually, 28 participants dropped out of the intervention group, compared with 20 in the control group (Figure 1).

The final sample size (n=588) included for data analysis was large enough to enable the detection of a 20% difference in the percentage of participants with blood pressure under control between the 2 groups. A national survey showed that about 38% of hypertensive patients had their blood pressure well controlled,^42^ but the government aimed for a 60% goal.^43^ A sample of 160 participants in each group would be required to detect the significance of such an achievement at α=0.05 and β=0.80 according to the formula below:

$$N=\frac{{\left[Z_{\alpha }\sqrt{2P(1-P)}+Z_{\beta }\sqrt{P_{c}(1-P_{c})+P_{e}(1-P_{e})}\right]}^{2}}{{\left(P_{c}-P_{e}\right)}^{2}}$$

Where Z_α_ for α level is the corresponding standard normal, Z_β_ for β level is the corresponding standard normal, N is the required sample size for each group, and P_c_ and P_e_ represent the percentage of participants with their blood pressure under control in the control and the intervention groups, respectively; P= (P_c_+P_e_)/2.

The sample size was also large enough to enable detection of a very small effect size (0.1) for the continuous indicators (knowledge and medication adherence scores) while α being set at 0.05 and statistical power (1−β) being set at 0.8.^44^

### Randomization

The random allocation of study participants was conducted by the researchers who were not involved in the implementation of the trial. The eligible patients were sorted in order according to the time when they agreed to participate in the trial. They were divided into 2 groups using a table of random numbers, which involved 3 steps. First, a starting point was randomly identified in the table. Then a backward or forward direction was randomly determined to retrieve 636 random numbers from the identified starting point of the table. Finally, the random numbers were mapped with the participants in sequence, which were then reordered from small to large. The first 318 patients with smaller numbers were assigned to the control group, while the rest were assigned to the intervention group.

### Trial Protocol

The trial started in June 2018 and lasted for 6 months.

All the participants received the usual care and continued their community systematic management program delivered by a team comprising general practitioners, nurses, and public health workers. The systematic management program for hypertension was part of the National Essential Public Health Package being offered to all eligible community residents for free. Once every 3 months, follow-up with hypertensive patients was conducted at outpatient appointments, telephone interviews, and home visits by monitoring patients' blood pressure and heart rate; changes in symptoms, comorbidities, and critical complications; risk factors (such as body mass index, smoking, drinking, salt intake, and physical exercise); and medication compliance. The patients identified with poorly controlled conditions were supposed to be given further medical advice or referred to a specialist clinic if needed and followed up again 2 weeks later. The routine follow-up arrangement was complemented with an annual physical examination, including blood tests and electrocardiograms.^31^ In addition to the community systematic management program, patients also had the freedom to bypass community health services to seek medical attention from hospitals covered by social health insurance programs. By 2011, almost all Chinese citizens had been covered by social health insurance policies, albeit with varied benefit entitlements.^45^

This trial tested the effectiveness of additional interventions involving pharmacists on the control of blood pressure. Five registered pharmacists were recruited from local tertiary hospitals (in China, very few, if any, properly trained registered pharmacists work in community health services). All of them had a master's degree and had engaged in clinical practice for more than 8 years. They showed a strong willingness to participate in the trial and maintained high levels of commitment throughout the trial. The pharmacists conducted a medication review for each patient in the intervention group based on the data collected through the baseline survey. A medication adjustment plan, if needed, was then developed considering the safety, effectiveness, and adherence barriers. The medication adjustment plan was further reviewed and fine-tuned monthly by the pharmacists based on a chart report. The monthly medication chart was developed by the assistants (medical students) of the pharmacists through outpatient appointments, home visits, or telephone interviews, during which they recorded patient uptake and adherence to the prescriptions (dosage, timing, and frequency of medicines), self-recorded blood pressure (at least weekly), and patients' main symptom complaints. If necessary, pharmacists conducted additional telephone interviews to better understand the situation of the patients (such as those with missing information). Interviews lasted about 6–10 minutes. In total, no more than 5 additional phone interviews were conducted each month. There was not a fixed arrangement between a pharmacist and the patients. A patient might receive the intervention from different pharmacists (or/and their assistants) at different times.

The pharmacists informed both patients and their general practitioners of the proposed medication adjustments, but general practitioners were responsible for advising the patients about any medication changes. Meanwhile, the pharmacists also developed an individualized education plan for each of the patients with risk behaviors. The plan was conveyed to the patient orally by the pharmacists or their assistants. Three months after the start of the intervention, the pharmacists delivered a group presentation in each community health center for the participants in the intervention group. More than 80% of the patients in the intervention group attended the group presentations.

### Measurements

The intervention effect was measured by 3 indicators:

1. **Blood pressure**: Blood pressure was measured by a calibrated sphygmomanometer (Omron HBP-1300 electronic sphygmomanometer) and recorded as systolic/diastolic mmHg. Each participant was measured 3 times with a 5-minute interval. An average reading was recorded. For most patients including those with diabetes or/and chronic kidney diseases, a reading below 140/90 mmHg was deemed normal and achievable. But a reduction of blood pressure to lower than 150/90 mmHg for those aged over 65 years with no complications was also considered acceptable.^31^
2. **Hypertension knowledge**: Patient knowledge of hypertension was measured by a scale developed by the research team. The scale was adapted from 2 existing instruments^23^^,^^46^ and guided by the 2017 Chinese National Guidelines for Prevention and Control of Hypertension in Primary Care.^31^ The scale contained 10 items, measuring the definition, risk factors, potential complications, and available interventional strategies of hypertension. A correct answer was given a score of 1, otherwise 0. Four of the items contained more than 1 correct answer. This resulted in a summed score ranging from 0 to 32. The summed score was transformed into a percentage score, with a higher score indicating higher knowledge and >60% being considered “pass.” Consultations were sought from 10 experts specialized in medicine, pharmacy, and public health on the content validity of the scale. The scale had a Cronbach's α of 0.919, indicating excellent internal consistency.
3. **Medication adherence**: Medication adherence refers to the degree to which a patient implements the medication treatment regimen. It was assessed using a validated 8 item scale developed by Morisky (MMAS-8).^47^ Respondents were asked to answer “yes” (1) or “no” (0) to the first 7 items. The last item was rated on a 5-point Likert scale ranging from 0 to 1. The scores were then summed, with a higher score indicating higher adherence. A score lower than 6 was deemed low adherence, while a score of 8 was considered high adherence. The MMAS-8 had a Cronbach's α of 0.786 in this study, indicating good internal consistency. In addition, the percentage of participants taking antihypertensive medications regularly and the percentage of participants with a treatment regimen with no need for adjustment were also calculated.

### Data Collection

The blood pressure of the participants was recorded before the trial (baseline, June 2018) and 3 and 6 months after commencement of the trial. Data about hypertension knowledge and medication adherence were collected twice through a questionnaire survey: once at the baseline and again at the end of the trial, which also included information about the sociodemographic characteristics (age, gender, and education), risk factors (smoking, drinking, exercise, and salt intake), duration of diagnosed hypertension, and coexisting chronic conditions of the respondents.

Participating pharmacists' assistants conducted household visits, measured blood pressure, and administered the questionnaire through face-to-face interviews. All the assistants were postgraduate or undergraduate medical students and were trained and assessed against the 2017 National Guidelines for Prevention and Control of Hypertension in Primary Care^31^ before interacting with patients. A quality control officer examined logical errors embedded in the returned questionnaires.

### Data Analysis

This was a comparative trial. Although it was not possible to conduct a blinded trial, data were not analyzed until the trial ended. Data were input into Epidata 3.1 and analyzed using Stata version 15.0. A *P* value of less than .05 was considered statistically significant.

The number and percentage of participants with different sociodemographic characteristics were described. Differences in the outcome indicators between the intervention and control groups were compared using independent sample t-tests for the continuous measurements or Chi-square tests for the categorical measurements. Paired t-tests or Chi-square tests were performed to examine changes in the outcome indicators over time. Difference-in-differences (DID) analyses were performed to provide additional evidence on the effects of pharmacist intervention based on a linear regression model (for continuous measurements) or logistic regression model (for categorical measurements), adjusting for potential variations in confounding factors including age, gender, education, lifestyle (smoking, drinking, salt intake, and physical exercises), duration of hypertension, and coexisting chronic conditions:

$$y_{\text{it}}(\mathrm{or}\;\text{Logit}\;y_{\text{it}})=\beta_{0}+\beta_{1}\cdot {\text{group}}_{\text{it}}+\beta_{2}\cdot {\text{time}}_{\mathrm{it}}+\beta_{3}\cdot {\text{group}}_{\mathrm{it}}\cdot {\text{time}}_{\mathrm{it}}+\varphi X_{\text{it}}+\varepsilon_{\text{it}}$$

Where y is the outcome indicator (dependent variable), i represents each individual, t is different times; time is a dummy variable with 0 indicating pre-trial and 1 indicating post-trial; group indicates the group allocation of participants (0=control, 1=intervention); X represents all the control variables; ε is a random error. The interaction effect between group and time (group multiplied by time) detects the effect of the intervention. An enter approach was adopted.

### Ethics Approval

The study protocol was approved by the Ethics Committee of Zunyi Medical University. The trial was registered with the Chinese Clinical Trials Registry (registration number: ChiCTR1900028368).

Before the trial and data collection, the study nature, objectives, and details were explained to the potential participants. Written informed consent was obtained from those who were willing to participate in the study. The participants were informed about their right to withdraw from the study, which would not result in any negative consequences on their services and treatment. They were assured of confidentiality.

## RESULTS

### Participant Baseline Characteristics

A total of 588 patients completed this study: 290 in the intervention group and 298 in the control group. The study participants had an average age of 65.98 years (standard deviation [SD]=9.48). More than half (57.31%) were female. The majority (52.72%) had only completed primary school education. About 18.71% were smoking, 26.02% were drinking, and 17.86% had heavy salt intake (>5 g a day^48^) at the time of the baseline survey. Approximately 86.73% reported regular daily exercise of moderate intensity, such as walking, housework, sports, and recreational activities (according to the WHO, these activities consume about 3–6 metabolic equivalent oxygen).^49^ About one-fifth (19.39%) of the participants reported coexisting chronic conditions. More than 30% of the participants had been living with hypertension for 10 or more years. But less than 10% had their blood pressure under control. There were no significant differences in the baseline characteristics between the intervention and control groups, except for age. The intervention group had a higher percentage of elderly (aged ≥65 years) participants, about 2.55 years older than the control group (*P*<.001) (Table 1).

**TABLE 1.** Baseline Characteristics of Study Participants in a Randomized Controlled Trial on Effect of Pharmacist Interventions on Management of Hypertension, China

	Intervention (n=290)	Control (n=298)
	No. (%)	No. (%)
Gender		
Male	115 (39.7)	136 (45.6)
Female	175 (60.3)	162 (54.4)
Age, years		
<65	81 (27.9)	136 (45.6)
65–79	197 (67.9)	139 (46.6)
≥80	12 (4.1)	23 (7.7)
Education		
Primary school	160 (55.2)	150 (50.3)
Middle school	81 (27.9)	83 (27.9)
High school	34 (11.7)	51 (17.1)
University	15 (5.2)	14 (4.7)
Smoking		
No	243 (83.8)	235 (78.9)
Yes	47 (16.2)	63 (21.1)
Drinking		
No	223 (76.9)	212 (71.1)
Yes	67 (23.1)	86 (28.9)
Regular daily exercise		
No	33 (11.4)	45 (15.1)
Yes	257 (88.6)	253 (84.9)
Salt intake		
Light	113 (39.0)	105 (35.2)
Medium	123 (42.4)	142 (47.7)
Heavy	54 (18.6)	51 (17.1)
Years of living with hypertension		
<10	195 (67.2)	210 (70.5)
11–20	75 (25.9)	71 (23.8)
>20	78 (6.2)	15 (5.0)
Not sure	2 (0.7)	2 (0.7)
Other chronic conditions		
Yes	64 (22.1)	50 (16.8)
No	226 (77.9)	248 (83.2)
Blood pressure (mmHg)^a^		
<120/80	10 (3.4)	6 (2.0)
120–139/80–89	62 (21.4)	72 (24.2)
140–159/90–99	118 (40.7)	141 (47.3)
160–179/100–109	72 (24.8)	63 (21.1)
≥180/110	28 (9.7)	16 (5.4)

a Blood pressure was presented as SBP/DBP and the higher grade prevails.

### Blood Pressure

Control of blood pressure improved over time in both groups (*P*<.005). However, a significantly higher percentage of participants in the intervention group had their blood pressure under control (<140/90 mmHg or <150/90 mmHg for those aged over 65 years with no complications) at the end of the trial compared with the control group (60.7% vs. 40.9%, *P*<.001), despite a lack of difference in the baseline (Table 2).

**TABLE 2.** Study Participants With Blood Pressure Under Control in a Randomized Controlled Trial on Effect of Pharmacist Interventions on Management of Hypertension, China

Time	Intervention, No. (%)	Control, No. (%)	*P* Value
Baseline	95 (32.8%)	92 (30.9%)	.62
Third month	136 (46.9%)	114 (38.3%)	.03
Sixth month	176 (60.7%)	122 (40.9%)	<.001
*P* value	<.001	.03	

**FIGURE 2:**
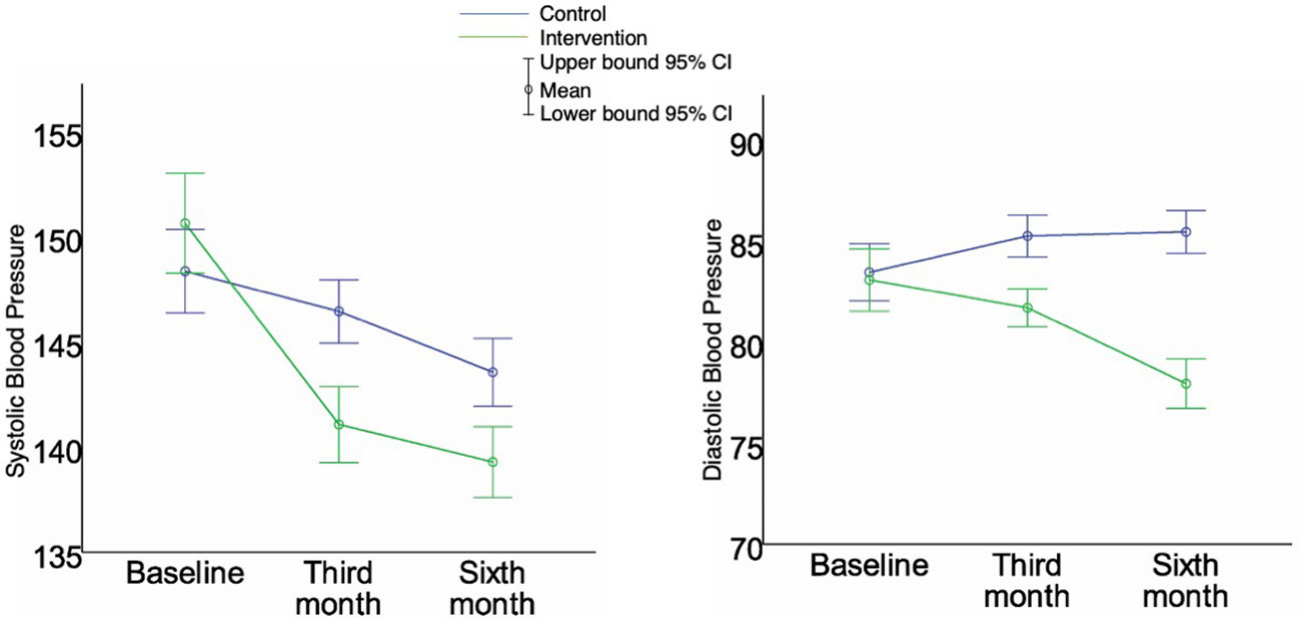
Changes in Systolic and Diastolic Blood Pressure Readings (mmHg) Among Participants in a Randomized Controlled Trial on Effect of Pharmacist Interventions on Management of Hypertension, China

The effects of the intervention became significant 3 months after the trial (46.9% vs. 38.3%, *P*=.034) and continued until the end of the trial (Figure 2).

Although there were no significant differences at baseline in systolic blood pressure (SBP) (*P*=.147) and diastolic blood pressure (DBP) (*P*=.713) readings between the intervention and control groups, significant differences appeared in both SBP (*P*<.001) and DBP (*P*<.001) 3 months after the trial. The gaps between the 2 groups remained at the end of the trial (*P*<.001) (Table 3). The intervention group ended up with significantly lower SBP and DBP readings than the control group. Both groups experienced a drop in SBP over time, but to a greater extent in the intervention group. The DBP reading declined in the intervention group, compared with a slight rise in the control group.

**TABLE 3.** Blood Pressure Readings of Study Participants in a Randomized Controlled Trial on Effect of Pharmacist Interventions on Management of Hypertension, China

		Intervention, mean±SD	Control, mean±SD	*P* Value
**Systolic blood pressure**	Baseline	150.61±20.44	148.34±17.33	.15
	Third month	141.07±15.52	146.44±13.24	<.001
	Sixth month	139.29±14.53	143.54±14.12	<.001
	*P* Value	<.001	<.001	
**Diastolic blood pressure**	Baseline	83.09±13.38	83.47±12.38	.72
	Third month	81.70±8.21	85.27±9.11	<.001
	Sixth month	77.94±10.51	85.47±9.29	<.001
	*P* value	<.001	.04	

Abbreviation: SD, standard deviation.

### Hypertension Knowledge

Hypertension knowledge scores increased over time in both groups (*P*<.001). However, significantly higher knowledge scores were found in the intervention group at the end of the trial compared with the control group (77.46±19.33 vs. 61.00±26.98, *P*<.001), despite a lack of difference in the baseline. At the baseline, about 37% of participants in the intervention group and 32% in the control group passed (scored over 60) the knowledge tests (*P*=.2). This increased to 84% and 51% in the intervention group and the control group, respectively, at the end of the trial (*P*<.001) (Table 4).

**TABLE 4.** Hypertension Knowledge of Study Participants in a Randomized Controlled Trial on Effect of Pharmacist Interventions on Management of Hypertension, China

	Knowledge Score, mean±SD			Participants with ≥60 Score, No. (%)		
	Intervention	Control	*P* Value	Intervention	Control	*P* Value
Baseline	47.08±27.94	43.35±27.84	.11	107 (37.20%)	96 (32.25%)	.20
Sixth month	77.46±19.33	61.00±26.98	<.001	244 (84.10%)	151 (50.70%)	<.001
*P* Value	<.001	<.001		<.001	<.001	

Abbreviation: SD, standard deviation.

### Medication Adherence

Significant differences in patient adherence to medications were found at the baseline between the intervention and control groups. Participants in the intervention group were more likely to take medications regularly (*P*<.001), adhere to prescriptions (*P<*.001), and have no need to adjust their treatment regimen (*P*=.02). Such differences remained at the conclusion of the trial (*P*<.001) although participants in both groups experienced significant improvement (*P*<.05) (Table 5).

**TABLE 5.** Antihypertensive Drug Treatment of Study Participants in a Randomized Controlled Trial on Effect of Pharmacist Interventions on Management of Hypertension, China

Antihypertensive Treatment		Intervention, No. (%)			Control, No. (%)			*P* Value for Group Difference	
		Baseline	Sixth Month	*P* Value	Baseline	Sixth Month	*P* Value	Baseline	Sixth Month
Administration of medications	Regularly	234 (80.7%)	277 (95.5%)	<.001	191 (64.1%)	210 (70.5%)	<.001	<.001	<.001
	Intermittently	43 (14.8%)	13 (4.5%)		48 (16.1%)	16 (5.4%)			
	Ignored	13 (4.5%)	0 (0%)		59 (9.8%)	72 (24.2%)			
Medication adherence score	Low (<6)	63 (21.7%)	13 (4.5%)	<.001	111 (37.2%)	89 (29.9%)	<.001	<.001	<.001
	Medium (6-7)	80 (27.6%)	14 (4.8%)		61 (20.5%)	18 (6.0%)			
	High (=8)	147 (50.7%)	263 (90.7%)		126 (42.3%)	191 (64.1%)			
Need for adjustment on treatment regimen	No	93 (32.1%)	184 (63.4%)	<.001	70 (23.5%)	91 (30.5%)	.03	.02	<.001
	Yes	197 (67.9%)	106 (36.6%)		228 (76.5%)	207 (69.5%)			

### Results of DID Analyses

The DID analyses confirmed that the intervention improved control of blood pressure significantly in terms of the 3 outcome indicators after adjustments for potential variations in confounding factors (Table 6). The intervention resulted in an increase in knowledge scores by 12.55 points (*P*<.001), a decrease in SBP readings by 6.65 mmHg (*P*=.001), and a decrease in DBP readings by 7.26 mmHg (*P*<.001) in comparison with the controls. The odds of participants passing hypertension knowledge tests in the intervention group was 4.45 times those in the control group (*P*<.001). Similarly, it was found that the intervention group had higher odds of not needing any adjustments of the treatment regimen (AOR=2.75, *P*<.001) and having their blood pressure under control (AOR=2.18, *P*=.002) compared with the control group.

**TABLE 6:** Difference-in-Differences Analyses on the Effects of Pharmacist Interventions on Study Participants' Blood Pressure

Outcome Indicator	Effect of Pharmacist Intervention					
Linear Regression Model for Continuous Measurement	Unadjusted β Coefficient			Adjusted β Coefficient^a^		
	Estimate	(95% CI)	*P* Value	Estimate	(95% CI)	*P* Value
Hypertension knowledge score	12.74	(12.56, 12.91)	<.001	12.55	(12.38, 12.71)	<.001
Systolic blood pressure reading	−6.52	(−6.63, −6.41)	.001	−6.65	(−10.39, −2.91)	.001
Diastolic blood pressure reading	−7.15	(−7.23, −7.08)	<.001	−7.26	(−9.27, −4.80)	<.001
**Logistic Regression Model for Categorical Measurement**	**Unadjusted Odds Ratio**			**Adjusted Odds Ratio** ^a^		
	**Estimate**	**(95% CI)**	***P* Value**	**Estimate**	**(95% CI)**	***P* Value**
% of patients with ≥60% hypertension knowledge score	4.13	(2.47, 6.94)	<.001	4.45	(2.60, 7.59)	<.001
% of patients with no need for adjustment on treatment regimen	2.57	(1.56, 4.24)	<.001	2.75	(1.64, 4.60)	<.001
% of patients with blood pressure under control	2.04	(0.35, 0.57)	.003	2.18	(1.33, 3.58)	.002

Abbreviation: CI, confidence interval.

a Adjustment for variations in gender, age, education, salt intake, smoking, drinking, physical exercise, duration of hypertension, and other chronic conditions.

## DISCUSSION

This study shows that community pharmacist interventions improve knowledge and medication adherence of hypertensive patients in the short term, which may have the potential to serve as an effective strategy for achieving the target control rate set by the Chinese government. Six months after the trial, 60.7% of the participants who received pharmacist interventions had their blood pressure under control, already exceeding the government target. In contrast, only 40.9% of the participants in the control group had their blood pressure under control, 19 percentage points below the governmental target. The marginal contribution of pharmacist interventions is high according to the DID analyses—more than doubling the odds of having blood pressure under control. This result is consistent with the findings of previous studies, although most were conducted in high-income countries.^50^^–^^53^ It shed some light on the hope in LMICs that hypertension can be controlled through community pharmacist intervention programs. Santschi and Colosimo^54^ concluded in a meta-analysis that better control of blood pressure can be achieved if pharmacists take a leading role in community interventions and engage in teamwork monthly. The intervention protocol tested in this trial aligns well with their proposal. However, our study did not test the long-term effect of the intervention, which warrants further studies.

Six months after the trial, 60.7% of the participants who received pharmacist interventions had their blood pressure under control, already exceeding the government target.

The study provides new insight into the potential role of pharmacists in the community control of hypertension in the Chinese health system context. It proved that pharmacist services in primary care can bring additional benefits, boosting the achievements of the existing medical doctor-led management program for hypertension. Two major mechanisms are likely to have enabled such an outcome.

First, pharmacists, as a profession that specializes in medications, are well positioned to educate patients on the appropriate use of medicines, which can be done through public education and individualized consultation services. This study adopted both approaches and showed that the knowledge score of the participants who received pharmacist interventions increased by 30 points, compared to less than 20 of those in the control group. This resulted in over 80% of participants passing the knowledge tests in the intervention group, more than thirty percentage points higher than that in the control group. There is evidence that the knowledge improvement had translated into better medication adherence as indicated by the higher increase in the percentage of participants in the intervention group taking regular medications and obtaining high medication adherence scores. International evidence shows that knowledge is indeed critical in helping hypertensive patients to better understand their conditions and adopt appropriate actions to control blood pressure.^55^^–^^57^ According to Amer and Nazir,^55^ pharmacists can help hypertensive patients reduce their concerns over the long-term use of medications, one of the major reasons for the intermittent use and ignorance of medications. It is worth noting that there was still space for further improvement of knowledge in the study participants when the trial concluded. Future studies should explore the knowledge attitudes belief behavior chain reactions of hypertensive patients.^58^^–^^59^

Second, pharmacists can assist medical doctors to assess and adjust medication regimens for hypertension. This study revealed that the need for the adjustment of drug therapy was very high before the commencement of the trial: 67.9% for the intervention group and 76.5% for the control group. This is not surprising given the low qualification profile of general practitioners in China. There is strong evidence suggesting that the timely adjustment of drug therapy is critical for the control of blood pressure.^60^ This study showed that the need for medication adjustments in the participants in the intervention group dropped from 67.9% to 30.5%, compared with a slight decline in the control group from 76.5% to 69.5%. The pharmacist interventions contributed to a more than doubling of the odds of not needing any medication adjustments in comparison with the control group. Unlike Hirsch's trial,^22^ this study did not allow pharmacists to initiate, adjust, or discontinue antihypertensive drug therapy independently. All changes had to be made on the advice of the general practitioners. In China, pharmacists have no prescription rights. They play an auxiliary role with a focus on dispensing prescribed medicines, which is not unique to China.^61^^–^^62^ Despite their restricted role, pharmacist interventions in this study achieved a similar effect size on the control of blood pressure compared to that in Hirsch's trial.^22^ Although we did not explore the underlying mechanism of the effect, it is clear that pharmacists could contribute to better management of hypertension. The possible reasons must be discussed in the context of the Chinese health system. There have been serious concerns in China that medical doctors are often overloaded and have failed to dedicate sufficient time to communicate with patients, let alone to engage patients.^63^ Clinical pharmacists played a bridging role in this trial. They not only communicated with and educated the patients but also liaised with medical doctors for better management of patients' blood pressure.

Pharmacists can assist medical doctors to assess and adjust medication regimens for hypertension.

It is important to note that as in many other LMICs, pharmacists in China are in short supply. Their functions are complementary to those of medical doctors, nurses, and public health workers. This study is by no means suggesting that pharmacists should bridge the shortfall of general practitioners in China. Pharmacist interventions have to be considered as an integral part of interdisciplinary team efforts for the community control of hypertension.^9^^,^^64^^–^^67^ In some countries, community pharmacists have been designated specific roles in managing chronic diseases.^23^^,^^64^^,^^68^^,^^69^ But China has yet to do so. By the end of October 2019, there were a total of 509,374 registered pharmacists in China, which is the equivalent of 3.7 pharmacists per 10,000 population.^70^ This level is very low compared to other countries. According to the World Federation of Pharmacists, 6.2 pharmacists per 10,000 population are recommended, although developed countries such as Australia and Canada have far more (10–20) pharmacists per 10,000 population.^71^ The gap in the supply of pharmacists is even greater than that of medical doctors.

A systems approach is needed to maximize the role and functions of pharmacists, in particular, to manage chronic diseases. The distribution of registered pharmacists in China is currently heavily concentrated in large metropolitan areas and large institutions. Very few, if any, pharmacists work in community health services. Although hospital pharmacists have started to increase their involvement in ward rounds and medication consultations, their main role is still dispensing prescribed medicines. In 2017, medication therapy management was written into the revised service specification for registered pharmacists for the first time in China.^72^ This signals a shift toward “patient-centered” pharmacist services. After many years, the Chinese government has started to call for an accelerated transformation of pharmaceutical care.^73^ This provides an opportunity to explore how pharmacist services can be incorporated into the most recent development of the integrated care network, in which large hospitals are encouraged to establish partnerships with primary care institutions to improve the continuity and coordination of patient care.^74^^–^^75^ This study recruited 5 hospital pharmacists to lead the pharmacist interventions, but we do not expect community health services to be able to employ registered pharmacists in the short term. Instead, we envisage a situation where community health services appoint and train some staff (not necessarily registered pharmacists) to serve as assistants to hospital pharmacists. Such an arrangement fits well into the mission and scope of integrated care networks in China.

A systems approach is needed to maximize the role and functions of pharmacists to manage chronic diseases.

The pharmacist education system in China needs to be reformed. University degree programs should place greater emphasis on patient-centered services to better prepare students for the expanded role of registered pharmacists. Meanwhile, large numbers of pharmacy workers (or pharmacist assistants as labeled in this study) need vocational and continuing education to fulfill a function beyond dispensing/sales of medicines. Community health services can also offer placement opportunities for medical and pharmacy students in senior years or at postgraduate levels to enhance their competency in managing chronic diseases through drug therapy. Serious challenges lie ahead. These changes not only require strong funding and policy support but also require recognition of the new identity of the pharmacist profession.^5^^,^^13^

To the best of our knowledge, this is the first randomized controlled trial in China testing the effects of community pharmacist interventions on the control of hypertension. We followed a strict protocol for this randomized control trial. The study participants were randomly selected and highly diversified, which indicates a potential for the results to be generalized to other groups of patients with hypertension. However, the pharmacists who conducted the interventions were highly selective. They were well respected due to their high degree qualification and work status (tertiary hospital). This would limit the potential of the results to be generalized. China has a serious shortage of clinical pharmacist workforce. It will be challenging to add further workloads on the already overloaded pharmacists.

### Limitations

This study has several limitations. First, the trial ended after 6 months due to resource restrictions. It did not test the long-term effect of the interventions. Second, this study did not perform detailed costing analyses on the interventions, but we recorded an average of 14 minutes of contact time per month from the pharmacists and their assistants for each participant in the intervention group. Third, the trial could not adopt double blindness in the design. Improvement in the control group was observed in this study, albeit at a smaller scale. We could not exclude the potential flowover effect when a medical doctor serves both patients in the intervention and control groups. This may lead to an underestimation of the effect size of the interventions. Fourth, a baseline difference in medication adherence appeared between the intervention and the control groups although the 2 groups of participants were randomly allocated. This was an unexpected result possibly due to the high diversity of patients with hypertension and the limited sample size. A stratified randomization strategy may generate a more balanced sample.

## CONCLUSION

The value of pharmacists may have been underappreciated. This study shows that pharmacists can make a significant contribution to improving community management of hypertension through educating patients and providing medication consultations with medical doctors, although the long-term effect remains unclear. This study demonstrated that community pharmacist interventions have significant short-term effects on improving the knowledge and medication adherence of hypertensive patients, as well as timely adjustments of drug therapy regimens from medical doctors, resulting in lowered blood pressure and increased control rate. However, more and better-educated pharmacists would be needed before such an initiative can be introduced. Equally important is the improvement of the entire care process centered around the need of patients. This often requires adjustments of roles and functions of various health professionals. There is a need to increase the public and policy makers' awareness about the important role of community pharmacists in managing chronic conditions. Further studies should assess the long-term effect and cost-effectiveness of such services.

## Notes

**Peer Reviewed**
